# Emerging Insights into the Interplay Between PANoptosis and Autophagy in Immune Regulation and Immune-Mediated Inflammatory Diseases

**DOI:** 10.3390/medsci13040310

**Published:** 2025-12-08

**Authors:** Ferenc Sipos, Györgyi Műzes

**Affiliations:** Immunology Division, Department of Internal Medicine and Hematology, Semmelweis University, H-1088 Budapest, Hungary

**Keywords:** PANoptosis, PANoptosome, autophagy, immune-mediated inflammatory diseases, innate immunity, adaptive immunity, systemic lupus erythematosus, rheumatoid arthritis, Sjögren’s syndrome, psoriasis, inflammatory bowel disease, inflammatory cell death

## Abstract

PANoptosis is an integrated form of regulated cell death that combines pyroptosis, apoptosis, and necroptosis through a coordinated molecular platform known as the PANoptosome. Autophagy, in parallel, maintains immune homeostasis by controlling cellular stress responses. Although both pathways are essential for innate and adaptive immunity, their functional interplay has only recently been explored. This review summarizes current knowledge on the bidirectional relationship between PANoptosis and autophagy, with emphasis on how autophagy can restrain PANoptotic signaling or, under certain conditions, promote inflammatory cell death. We discuss cell-type-specific aspects of this crosstalk in macrophages, dendritic cells, monocytes, neutrophils, T cells, and B cells, focusing on key PANoptosis mediators and autophagy-related proteins. We then examine how dysregulated autophagy and exaggerated PANoptotic signaling contribute to chronic inflammation and tissue damage in immune-mediated inflammatory disease, including systemic lupus erythematosus, rheumatoid arthritis, Sjögren’s syndrome, psoriasis, and inflammatory bowel disease. Finally, we outline shared molecular principles that position the autophagy–PANoptosis axis as a fundamental immunoregulatory mechanism and a promising source of therapeutic targets in chronic inflammatory and autoimmune disorders.

## 1. Introduction

Pyroptosis, apoptosis, and necroptosis are distinct forms of regulated cell death that converge in a unified mechanism termed PANoptosis [1]. This process is coordinated by the PANoptosome, a multiprotein complex that integrates these pathways to drive inflammatory cell death [1]. PANoptosis plays a central role in innate immunity by enabling rapid elimination of infected or damaged cells and initiating strong inflammatory responses [2]. Pathogen sensing through interferon-induced molecules such as Z-DNA binding protein 1 (ZBP1) or inflammasomes can trigger PANoptosome formation, resulting in cytokine (e.g., interleukin [IL]-1β, IL-18) and damage-associated molecular patterns (DAMP) release, which promote immune activation [1,3].

Beyond innate immunity, PANoptosis influences adaptive responses by modulating antigen-presenting cells, thereby shaping T and B cell activation [4,5]. The release of inflammatory mediators and neoantigens further enhances adaptive immunity, particularly during infections and in tumor settings [5,6].

Autophagy is another fundamental process in immune regulation [7]. It maintains cellular homeostasis, contributes to pathogen clearance through xenophagy [8], and limits excessive inflammation by downregulating inflammasome activity and modulating pattern recognition receptor signaling pathways [8]. Autophagy also fine-tunes cytokine production and interferon responses [9]. Autophagy-dependent cell death is a distinct type of regulated cell death in which the autophagic machinery is actively involved in the process of cellular death, rather than merely functioning as a survival mechanism [10,11]. Experimental models demonstrate that excessive or dysregulated autophagic flux—particularly involving the activation of Beclin-1, ATG5, and ATG7—can directly induce cell death pathways, highlighting that autophagy may function as both a protective and a cytotoxic mechanism depending on the context [12,13].

In adaptive immunity, autophagy facilitates MHC class II–mediated antigen presentation [14], including the display of endogenous antigens to CD4^+^ T cells, supporting antitumor and autoimmune responses [15]. It also contributes to lymphocyte development, function, and memory maintenance [15]. Together, PANoptosis and autophagy form a tightly interconnected network that balances host defense, inflammation, and immune regulation.

A recent study has shown that autophagy and PANoptosis are functionally interdependent [10]. Research indicates a connection and interaction between these two processes, which either promote or counterbalance each other [16]. Autophagy degrades PANoptosome components or removes danger signals such as mitochondrial reactive oxygen species (ROS) and cytosolic DNA to decrease PANoptosis. In contrast, autophagy may enhance stress responses or modulate cytokine production to aid PANoptotic signaling [10,16]. These pathways interact dynamically, affecting immune cell fate decisions, especially during infection, tumor growth, or chronic inflammation. The immune system must balance tolerogenic survival signals and proinflammatory cell death to maintain homeostasis and prevent immunopathology. Disruption of this balance can cause immunodeficiency or excessive immune activation in autoimmune and autoinflammatory illnesses. Despite rising understanding of this interaction, the autophagy-PANoptosis axis’ molecular underpinnings are still unclear. Understanding how these pathways interact in diverse immune cell types and disease situations is essential for developing new immunological balance therapies.

This article explains the intricate and poorly understood relationship between autophagy and PANoptosis. We aim to integrate how these interconnected pathways affect immune function and pathology by studying their molecular intersections across innate and adaptive immune cells and their dysregulation in immune-mediated inflammatory diseases (IMIDs).

## 2. The Overview of PANoptosis and Autophagy

Deciphering IMID pathophysiology requires understanding immune homeostasis and inflammatory response molecular pathways. PANoptosis and autophagy are key systems that regulate cell fate and immunity. This section briefly discusses PANoptosis and autophagy due to their importance and growing evidence of their interaction. We hope to understand how deregulation of these mechanisms causes autoimmunity by describing their main molecular components and regulatory pathways. This overview prepares for future sections on their interaction and implications in various autoimmune contexts.

### 2.1. PANoptosis

Programmed cell death is traditionally classified into the following types: intrinsic apoptosis, extrinsic apoptosis, mitochondrial permeability transition-driven necrosis, necroptosis, ferroptosis, pyroptosis, parthanatos, entotic cell death, NETotic cell death, lysosome-dependent cell death, autophagy-dependent cell death, and immunogenic cell death (ICD) [17]. A recent study identified a novel, integrated form of cell death that combines the mechanisms of pyroptosis, apoptosis, and necroptosis, termed PANoptosis [1]. The PANoptosome, a multifunctional protein complex, orchestrates PANoptosis by simultaneously activating caspases, gasdermin D, and necroptotic kinases [18].

The activation of PANoptosis results from the coordinated action of numerous molecules. The PANoptosome is a supramolecular signaling complex that integrates critical components of the apoptotic, pyroptotic, and necroptotic pathways [19]. Its assembly is triggered by pathogen-associated molecular patterns (PAMPs), DAMPs, or cytokine signaling (e.g., tumor necrosis factor [TNF], interferons [IFNs], and IL-1) [20].

The assembly of the PANoptosome primarily depends on scaffold proteins such as ZBP1, apoptosis-associated speck-like protein containing a CARD (ASC), and receptor-interacting serine/threonine-protein kinase 3 (RIPK3) [21,22]. Upon formation, the PANoptosome recruits and activates various cell death effectors, including caspases (CASP1, -3, and -8), gasdermins (GSDMD, GSDME), and mixed lineage kinase domain-like pseudokinase (MLKL), leading to the coordinated execution of cell death pathways [23].

While ZBP1, together with caspases, RIPK3, and gasdermins, is a well-established upstream regulator of PANoptosis in several viral and sterile inflammation settings, different PANoptosis-inducing contexts can rely on distinct sensors and scaffolds, and not all forms of PANoptosis are strictly ZBP1-dependent [24]. Caspase-1 cleaves the proinflammatory cytokines IL-1β and IL-18, as well as GSDMD, when activated by the inflammasome, resulting in the formation of pyroptotic pores [24]. Caspase-3 and caspase-7 are the canonical executioners of apoptosis, cleaving substrates such as poly (ADP-ribose) polymerase (PARP) [25]. Caspase-8 functions at the intersection of necroptosis and apoptosis: it can promote apoptosis by either inhibiting necroptosis through suppression of RIPK3/MLKL activation, or by directly initiating apoptosis via death receptor signaling [25].

RIPK3 is a key regulator of necroptosis that interacts with RIPK1 and MLKL [26]. It phosphorylates MLKL, which leads to plasma membrane rupture and necroptotic cell death, especially in the absence or inhibition of caspase-8 [27]. In PANoptosis, RIPK3 interacts with ZBP1, facilitating the formation of the PANoptosome [28]. Membrane pore formation, a hallmark of pyroptosis, results from GSDMD cleavage by caspase-1 or caspase-11. GSDME is cleaved by caspase-3, enhancing inflammatory responses and converting apoptosis into secondary pyroptosis [29].

ZBP1 is a central upstream sensor that initiates PANoptosis by recognizing endogenous or pathogen-derived Z-nucleic acids and integrating type I interferon (IFN) signals into inflammatory cell-death programs [30]. Upon activation, ZBP1 recruits RIPK3 through its RHIM domains, enabling the assembly of a multi-protein PANoptosome complex that incorporates RIPK1, caspase-8, and inflammasome components [31,32]. Through these interactions, ZBP1 simultaneously drives apoptotic, pyroptotic, and necroptotic pathways, positioning it as a uniquely potent coordinator of inflammatory cell death [31,32,33,34]. Autophagy plays an important modulatory role by limiting the availability of mitochondrial DAMPs and cytosolic nucleic acids that otherwise potentiate ZBP1 activation; therefore, impaired autophagy creates a cellular environment that lowers the threshold for ZBP1-dependent PANoptosis [35]. Type I IFN signaling further amplifies this process by upregulating ZBP1 expression, making IFN-rich inflammatory diseases particularly susceptible to ZBP1-mediated PANoptotic responses [31,36]. Overall, ZBP1 functions as a stress- and nucleic-acid–responsive molecular switch linking innate immune sensing, inflammasome activation, and regulated cell-death pathways within the PANoptosis framework.

#### PANoptosis as a Form of Immunogenic Cell Death

Recent evidence increasingly indicates that PANoptosis represents a highly immunogenic type of regulated cell death, exhibiting several characteristics similar to classical immunogenic cell death (ICD). PANoptotic cells release or expose various potent DAMPs, such as High-mobility group box 1, adenosine triphosphate, mitochondrial DNA, S100 proteins, and IL-1 family cytokines, which can activate dendritic cells and enhance adaptive immune responses [13,17,37,38]. The PANoptosome components, including ZBP1, Absent in melanoma 2 (AIM2), and caspase-8, facilitate significant inflammasome activation and enhance the release of IL-1β and IL-18, which are critical cytokines associated with ICD. Gasdermin-mediated membrane permeabilization promotes the extracellular release of DAMPs, thereby increasing the immune visibility of dying cells [29,39]. PANoptosis is linked to the release of mitochondrial DAMPs and oxidized mtDNA, which significantly activate type I interferon pathways—another key axis of ICD [40,41,42]. Additionally, PANoptotic cell death leads to the presentation of calreticulin-like “eat-me” signals via ER–phagy and membrane stress pathways, thereby facilitating effective antigen uptake [12,13].

These findings suggest that PANoptosis incorporates various features characteristic of ICD, and its potential for eliciting an immune response likely surpasses that of apoptosis, pyroptosis, or necroptosis individually. Identifying PANoptosis as an inherently immunogenic process could enhance our comprehension of inflammatory diseases and facilitate the utilization of PANoptosis-derived DAMPs in immunomodulatory treatments.

### 2.2. Autophagy

Autophagy is an evolutionarily conserved intracellular degradation pathway that maintains cellular homeostasis by removing damaged organelles, misfolded proteins, and intracellular pathogens [43]. It is essential for stress adaptation, immune regulation, development, and aging, while its dysregulation contributes to cancer, neurodegeneration, infections, and autoimmune diseases [44].

Three major forms of autophagy are recognized: macroautophagy, microautophagy, and chaperone-mediated autophagy [43]. Macroautophagy (hereafter referred to as autophagy) is the dominant mechanism, involving the formation of double-membrane autophagosomes that deliver cytoplasmic cargo to lysosomes for degradation, whereas microautophagy involves direct lysosomal engulfment, and chaperone-mediated autophagy selectively translocates targeted proteins across the lysosomal membrane.

Autophagy is controlled by a coordinated network of autophagy-related genes (*ATGs*) [10]. The ULK1 complex initiates autophagosome formation, the Beclin 1-Vps34 containing class III PI3K complex I drives phagophore nucleation, and LC3 lipidation enables membrane elongation and cargo recruitment through adaptor proteins such as p62/SQSTM1. The nutrient-sensing kinase mTOR is the central negative regulator; stressors such as nutrient deprivation, oxidative stress, or infection inhibit mTOR, thereby activating autophagy, whereas nutrient abundance suppresses autophagic flux [45].

The autophagic pathway proceeds through a streamlined sequence: ULK1 activation, phagophore nucleation by Beclin 1–Vps34, LC3-dependent elongation and autophagosome closure, and finally autophagosome–lysosome fusion followed by cargo degradation and metabolic recycling [11]. This tightly controlled process is essential not only for cellular quality control but also for shaping inflammatory signaling, placing autophagy at the core of immune regulation.

In addition to these canonical forms, several selective autophagy pathways -such as mitophagy, ER-phagy, and xenophagy- play essential roles in regulating immune signaling. These selective processes are discussed in later sections where they emerge as key modulators of ICD and IMIDs [10,43,45].

## 3. Mechanisms Influencing the Relationship Between Autophagy and PANoptosis

Cytoprotection and homeostasis are autophagy’s main tasks. It can regulate or promote programmed cell death, including PANoptosis, under certain situations [46]. Autophagy’s effects vary on cell type, initial stressor (e.g., infection, reactive oxygen species, hypoxia, DNA damage), and activation timing (acute vs. chronic) [47,48,49].

To improve readability, the mechanistic interactions between autophagy and PANoptosis are summarized below in a streamlined format highlighting the main regulatory steps.

Autophagy inhibits PANoptosis through several coordinated mechanisms:

(a) Degradation of PANoptosome components, such as AIM2, ZBP1, or pyrin via p62/SQSTM1-mediated selective autophagy [10,16,50,51].
(b) Removal of active inflammasomes, including NLRP3, thereby limiting caspase-1 activation and pyroptosis [52];
(c) Regulation of necroptotic effectors (i.e., RIPK1, RIPK3, and MLKL) through lysosomal degradation, preventing PANoptosome assembly [53];
(d) Attenuation of caspase-8 activity, indirectly reducing apoptosis and limiting integration of death pathways [54].

Together, these steps illustrate how autophagy functions as a negative regulator of PANoptosis. Most mechanistic evidence for the degradation of inflammasome and PANoptosome components in *ATG5-*, *ATG7-* or *ATG16L1*-deficient settings derives from murine macrophages and dendritic cells, whereas direct confirmation in human primary immune cells is still limited.

Under specific inflammatory or infectious conditions, autophagy may instead facilitate PANoptosis:

(a) Autophagy dysfunction leads to enhanced pro-apoptotic signaling and caspase-8 activation [55,56,57];
(b) Impaired mitophagy promotes ROS accumulation and mtDNA release, amplifying inflammasome and PANoptosome activation [58,59,60];
(c) Cytokine-rich microenvironments (e.g., type I IFN, IL-1β) shift autophagy from a cytoprotective to a pro-death role [12,16,40,61];
(d) Viral infections may exploit autophagy to enhance ZBP1-dependent PANoptotic signaling [5,62].

These findings underscore the context-dependent duality of autophagy in PANoptosis regulation. These pro-PANoptotic effects of impaired autophagy have been primarily demonstrated in murine viral infection models, particularly influenza-induced ZBP1 activation, with human data being more indirect.

### 3.1. How Does Autophagy Influence the Regulation of PANoptosis Across Distinct Immune Cell Populations and Under Varying Stress Conditions?

#### 3.1.1. Autophagy and PANoptosis in Innate Immune Cells

Innate immune cells interact with autophagy and PANoptosis on multiple levels. Often, these two processes are antagonistic. Autophagy increases cell survival and has anti-inflammatory effects, while PANoptosis is an inflammatory, cascade-like cell death that combines pyroptosis, apoptosis, and necroptosis.

Autophagy typically mitigates PANoptosis through several mechanisms:

(a) elimination of DAMPs and PAMPs, such as lipopolysaccharide (LPS), mitochondrial ROS, and cytosolic DNA [10,37,63];
(b) mitophagy limits NLRP3 activation by clearing damaged mitochondria [64];
(c) macroautophagy-mediated degradation of NLRP3 inflammasomes [64]; and
(d) inhibition of the release of inflammatory mediators, such as IL-1β [16,65].

Several biochemical connections have been identified between autophagy and PANoptosis. The PANoptosis activator ZBP1 is induced in innate immune cells -particularly macrophages and dendritic cells- in response to viral infection or interferon signaling, promoting PANoptosome assembly and activation [66,67,68,69]. In the absence of functional autophagy, ZBP1 expression is amplified, enhancing PANoptosis via the activation of RIPK3, caspase-8, and gasdermin D [16].

Caspase-8 regulates autophagy by cleaving ATG3, activates non-canonical inflammasome pathways, and contributes to apoptotic signaling [70,71,72,73,74]. Thus, caspase-8 serves as a molecular switch at the intersection of survival and death pathways [24,70,71,72,73,74,75,76].

The initiation and progression of autophagy need ATGs such as ATG5, ATG7, and ATG16L1 [77,78]. Knockout studies show that genetic loss of these proteins causes excessive inflammasome activation, PANoptosis in macrophages and dendritic cells, and elevated IL-1β and IL-18 production [3,79,80,81,82,83,84,85].

Finally, mitophagy acts upstream to restrain NLRP3 activation and modulates PANoptotic signaling by targeting damaged mitochondria and suppressing mitochondrial-derived DAMPs [16]. Table 1 summarizes context-dependent autophagy-PANoptosis cross-talk outcomes. The depth of mechanistic insight varies among immune cell types; while macrophages and neutrophils have well-characterized autophagy–PANoptosis interactions, evidence in dendritic cells, monocytes, and NK cells remains more limited and often indirect. Notably, these observations mainly originate from *Atg5-, Atg7-* or *Atg16l1*-deficient murine macrophages and dendritic cells, whereas equivalent studies in human cells remain comparatively scarce.

#### 3.1.2. Macrophages

During influenza virus infection, macrophages activate ZBP1 when autophagy fails to remove damaged organelles or viral components. This defect helps form the PANoptosome, a ZBP1-NLRP3-caspase-8-RIPK3 complex [41,64,86]. Deleting or functionally losing autophagy genes such as *ATG5* or *ATG7* boosts inflammasome activation and PANoptosis [80,87]. ROS accumulate due to reduced autophagic flux, activating the NLRP3 inflammasome and inducing PANoptosis [88,89,90].

PANoptosis’ central adapter and autophagy regulator is caspase-8. This is done by targeting core autophagy proteins such as ATG3 and ATG5 for proteolytic destruction [70,91]. Autophagy modulates ZBP1 and RIPK3 expression and activity, affecting PANoptosis [86].

In summary, macrophages represent one of the best-characterized systems in which impaired autophagy directly lowers the threshold for ZBP1–RIPK3–caspase-8-driven PANoptosis. Most evidence linking ZBP1 to PANoptosome assembly in response to viral infection comes from murine bone-marrow-derived macrophages, with only limited parallel data available from human systems.

Baicalin, a flavonoid from *Scutellaria baicalensis*, has been shown to modulate autophagy and inflammatory cell death pathways in macrophages [92,93,94,95]. Its effects include activation of PI3K/Akt/mTOR-dependent autophagy and suppression of NLRP3-driven inflammatory cytokine release. These findings suggest that flavonoids may modulate the autophagy–PANoptosis axis, although their therapeutic relevance requires broader contextual evaluation [96,97].

PANoptosis is linked to mitochondrial malfunction and Z-DNA accumulation [22]. Baicalin prevents mitochondrial damage and Z-DNA production, inhibiting the ZBP1-containing PANoptosome complex from assembling in macrophages [22]. Baicalin inhibited PANoptotic signaling in liver-resident macrophages (Kupffer cells) in an in vivo hemophagocytic lymphohistiocytosis model, reducing systemic inflammation and protecting organs [22].

#### 3.1.3. Dendritic Cells

Autophagy aids dendritic cell (DC) maturation, including antigen presentation, migration, and cytokine release [98]. TLR signaling pathways are also regulated by it [98,99]. The PANoptosome multiprotein complex contains the inflammasome sensor NLRP3 [100]. DCs are primed for inflammasome activation by TLR signaling [101]. Activating TLR4 increases the synthesis of pro-IL-1β, which is then converted into mature IL-1β by the inflammasome [102]. This may cause pyroptosis.

TLRs provide priming signals that activate the inflammasome and PANoptosome in dendritic cells during sterile inflammation or infection [101]. Thus, DCs’ innate immunological response involves inflammasome activation and PANoptosome formation, controlled indirectly by TLR signaling.

Overall, while direct mechanistic evidence remains limited, current data indicate that autophagy indirectly shapes PANoptosis susceptibility in dendritic cells through its effects on TLR-dependent priming and inflammasome activity.

#### 3.1.4. Monocytes

ROS can activate monocyte autophagy, especially in stressed or injured cells [103]. THP-1 cells induce autophagy in response to hemin-generated ROS [104]. Autophagy degrades damaged mitochondria, the main intracellular source of ROS [13].

ROS regulate autophagy via mTOR and stimulate AMP-AMPK, which inhibits mTOR and increases autophagy [105].

Monocyte behavior and differentiation are affected by autophagy, which is modulated by ROS. Furthermore, autophagy has the ability to both inhibit and promote the synthesis and secretion of IL-1β [98]. One possibility is that pro-IL-1β can be destroyed in autophagosomes, reducing secretion [62,106]. Alternatively, autophagy may cause the abnormal release of mature IL-1β by directing it to autophagosome-derived vesicles for export [107].

Inflammatory cell death can result from monocyte PANoptosome activation [108]. This activation can be caused by inflammatory mediators and microbial pathogens [108,109]. In monocytes, autophagy–PANoptosis interactions are inferred largely from inflammasome activation and ROS-driven stress pathways rather than direct PANoptosome characterization.

Experimental support for ROS-driven autophagy–inflammasome interactions in monocytes comes from both human THP-1 monocytic lines and murine models, though PANoptosis-specific evidence remains largely inferred.

#### 3.1.5. Neutrophils

Autophagy and lipophagy are strongly linked in neutrophil biology from the early stages of development [110]. While myeloperoxidase release is unaltered, autophagy deficiency hampers neutrophil degranulation, especially primary and secondary granules [110]. Serum fasting or rapamycin therapy activates autophagy pathways, including xenophagy and LC3-associated phagocytosis, to remove adherent-invasive *Escherichia coli* [111]. Intravenous immunoglobulin induces autophagy, which boosts bactericidal efficacy against multidrug-resistant bacteria [112].

Neutrophil extracellular trap (NET) production requires autophagy beyond microbial death. Autophagy and ROS are needed for chromatin decondensation, a critical NETosis step [113]. Inhibiting either route switches NETosis to apoptosis. While the ROS-autophagy axis is crucial to NET formation, autophagy can be induced by both ROS-dependent and ROS-independent pathways [113,114]. Autophagy in neutrophils protects against infection by better microbial clearance, but it can potentially worsen pathological inflammation by increasing excessive NET formation and cytokine release, especially under certain inflammatory settings.

Gasdermin D in neutrophils promotes psoriasis-like inflammation, although GSDMD reduction reduces proinflammatory cytokine release [115,116,117,118]. Neutrophil pyroptosis drives inflammation [115,116,118]. Cleavage of GSDMD at aspartic acid 88 did not suppress pyroptosis or cytokine release during canonical or non-canonical inflammasome activation in neutrophils or macrophages [39].

Caspase-8 regulates a constitutively active inflammatory pathway in neutrophils, requiring tonic IFN-β production and RIPK3, but not MLKL. Inhibiting caspase-8 boosts chemokine synthesis and neutrophil migration to inflammation [119]. Caspase-8-mediated GSDMD activation strengthens antibacterial defenses but also causes systemic inflammation and TNF-induced shock [39,120]. The caspase-8–GSDMD axis may contribute to a neutrophil PANoptosis-like cell death pathway, underlining its complicated and context-dependent roles in inflammation and host defense [39].

#### 3.1.6. Natural Killer Cells

A recent study found that robust autophagic activity is needed for maturation of immature natural killer (NK) cells [121]. NK cell death results from autophagy deficiencies, which cause mitochondrial malfunction and increased ROS. Autophagy removes damaged mitochondria and limits ROS to keep NK cells alive during development. FoxO1 is a crucial regulator of this process. FoxO1 in the cytoplasm of immature NK cells (iNKs) initiates autophagy via interacting with Atg7. Genetic deficit or production of an inactive *FoxO1*(AAA) mutant hampers autophagy start in iNKs, affecting NK cell growth and antiviral responses. These findings demonstrate the importance of FoxO1-mediated autophagy in NK cell development and innate immunity [121].

PANoptosis and autophagy in NK cells are still being studied. In NK cells, direct PANoptotic mechanisms have not yet been delineated, and current data reflect indirect links through mitochondrial stress, autophagy dependence, and caspase-8/RIPK3 signaling. Table 2 highlights innate immune cell autophagy and PANoptosis activities.

### 3.2. Autophagy and PANoptosis in Adaptive Immune Cells

Multiple, linked pathways of autophagy shape the adaptive immune response. It helps T lymphocytes digest and present antigens intracellularly, delivering them to major histocompatibility complex (MHC) molecules needed for T cell identification and activation [122,123]. Autophagy regulates T and B lymphocyte survival, homeostasis, and differentiation beyond antigen presentation, assuring immune cell development and function [122,124]. Mitophagy helps immune cells maintain mitochondrial integrity and function, adjusting their reactivity to external stimuli and bioenergetic needs [125]. Autophagy sustains energy and biosynthetic demands during differentiation in activated T cells, enabling metabolic reprogramming for clonal growth and effector function [126]. Autophagy also protects against autoimmunity by preventing excessive inflammatory reactions and maintaining self-tolerance [127]. Autophagy enhances CD8^+^ T cell responses by enhancing effective antigen processing and presentation, and by increasing dendritic cell capacity to activate cytotoxic T lymphocytes [15]. The regulation of CD4^+^ T cell-mediated immunity is influenced by autophagy, which modulates peripheral responses to pathogens and influences the selection and survival of these cells throughout thymic development [128]. Autophagy limits inflammation and regulates immunological homeostasis in addition to adaptive immunity. Degrading proinflammatory signaling intermediates and resolving inflammatory processes help it do this [84].

In cancer immunotherapy and infectious illnesses, PANoptosis is becoming an important immune response regulator in T cells [129,130]. PANoptosis dysregulation can impair T cell function, the ability to kill cancer cells or combat infections, and pathogen immune evasion [129,130]. PANoptosis in B cells is being studied in autoimmune disorders and cancer [131].

Autophagy-PANoptosis interaction in adaptive immune cells is a promising field of research with major implications for immune cell survival and function. In T and B lymphocytes, autophagy promotes cellular homeostasis and prevents premature or excessive inflammatory cell death [16]. Deficits in autophagy-related genes, such as ATG5 or ATG7, can decrease T cell survival and lower numbers, especially in CD4^+^ and CD8^+^ subsets. Oxidative stress and mitochondrial dysfunction may activate inflammasome or PANoptosome signaling pathways [132,133,134] and cause this impairment [135].

Innate immune signaling is increasingly linked to mitochondrial damage, bridging metabolic imbalance and inflammatory cell death. Activated T cells also express caspase-8 and RIPK3, which affect the fate of cells in inflammatory circumstances [136]. While full activation of the PANoptotic cascade is rare under physiological conditions, type I interferons or viral infections (e.g., lymphocytic choriomeningitis virus) can induce ZBP1 expression in T cells, sensitizing them to PANoptosis [30,137,138].

Autophagy helps B cells retain memory B cell populations for long-term humoral immunity [139]. ATG16L1 deficiency in macrophages is associated with increased IL-1β production, which can impact immunological responses and B cell function [14,140]. Though direct evidence for PANoptosis in B cells is limited, experimental data suggest that under stress conditions like hypoxia or viral challenge, components of the inflammasome and RIPK3/caspase-8 pathways may be activated in vitro, suggesting that B cells may induce PANoptotic mechanisms under certain pathological conditions [141,142]. In adaptive immune cells, autophagy and PANoptosis interact as shown in Table 3.

### 3.3. A Unified Mechanistic Model Integrating Autophagy and PANoptosis

**Figure 1:**
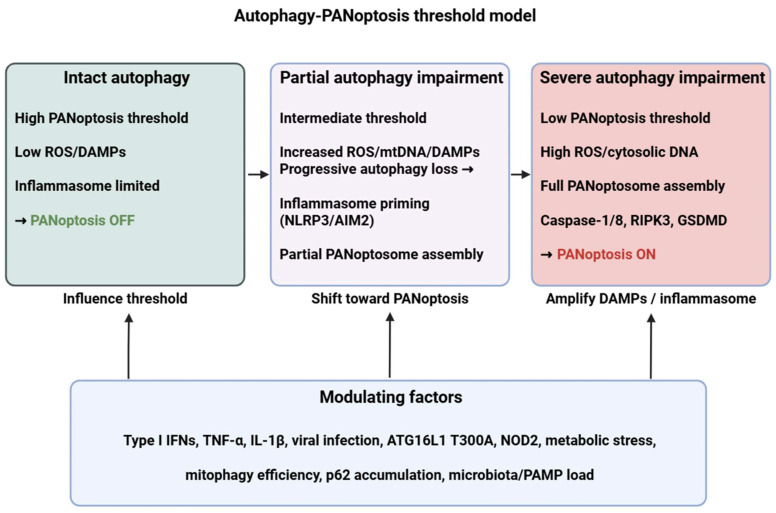
Autophagy–PANoptosis threshold model. Intact autophagy maintains a high activation threshold for PANoptosis by removing damaged organelles and inflammasome components. Progressive autophagy impairment (due to genetic variants, infection, cytokine milieu or metabolic stress) increases ROS/DAMPs and primes inflammasomes (NLRP3/AIM2), lowering the threshold for PANoptosome assembly (ZBP1/AIM2/ASC/caspase-8/RIPK3) and eventually engaging PANoptosis (caspase-1/8, RIPK3, GSDMD). ROS: reactive oxygen species; DAMP: danger-associated molecular pattern; mtDNA: mitochondrial DNA; NLRP3: NLR family pyrin domain containing 3; AIM2: Absent in melanoma 2; RIPK3: Receptor-interacting protein kinase 3; GSDMD: Gasdermin D; IFN: interferon; TNF: tumor necrosis factor; IL: interleukin; ATG16L1: autophagy related 16 like 1; NOD2: nucleotide binding oligomerization domain containing 2; PAMP: pathogen-associated molecular pattern. Figure was partly created with https://biorender.com/ (accessed on 4 December 2025).

To address the heterogeneous and context-dependent observations described above, we propose a unified “autophagy–PANoptosis threshold model” that synthesizes how these pathways interact across immune cell types and tissues (Figure 1). This framework explains (i) how autophagy modulates PANoptosis at shared upstream nodes, (ii) which hierarchical or conditional relationships determine whether autophagy suppresses or promotes PANoptotic signaling, and (iii) how disease-specific microenvironments shift these thresholds.

First, at the sensor level, autophagy and PANoptosis share multiple upstream danger-recognition nodes, including mitochondrial stress, cytosolic DNA and RNA, ER stress, and cytokine signaling (TNF, IFN-I/II). Autophagy acts as an early filter by removing damaged mitochondria, aggregated proteins, or inflammasome intermediates, thereby controlling the amount and duration of DAMP and inflammasome signals that also drive PANoptosome assembly. This shared-sensor architecture provides an explanation for why both pathways are activated by similar stressors across tissues.

Second, at the regulatory or threshold level, autophagy functions as a rheostat that determines whether PANoptotic signaling remains subthreshold (survival and controlled inflammation) or surpasses a critical activation point. Several conditional and hierarchical factors shape this threshold:

(1) autophagy capacity (basal vs. induced flux),
(2) mitochondrial quality control (mitophagy efficiency),
(3) inflammasome priming (e.g., type I IFN–dependent ZBP1 induction),
(4) cytokine milieu (TNF–IFN synergy), and
(5) metabolic state (mTOR–AMPK balance).

When autophagy effectively reduces DAMP burden, clears inflammasomes, and prevents sustained caspase-8/RIPK3 activation, PANoptosis is inhibited. Conversely, when autophagy becomes insufficient, overwhelmed, or pathologically hyperactivated, key regulatory switches—such as caspase-8 cleavage of ATG proteins or p62 accumulation—shift the balance toward PANoptosome assembly.

Third, at the outcome level, PANoptosis emerges only when threshold crossing is accompanied by specific inflammatory cues. Cells then engage an integrated pyroptotic–apoptotic–necroptotic program driven by ZBP1, RIPK3, caspase-8, ASC, and gasdermins. The model thereby explains why autophagy may either antagonize or facilitate PANoptosis depending on whether it limits or amplifies the upstream signals that converge on the PANoptosome scaffold.

Together, this unified model articulates a mechanistic consensus by which autophagy acts as a context-dependent gatekeeper of PANoptosis. It provides a conceptual scaffold that explains the dual regulatory roles observed experimentally and offers a basis for interpreting disease-specific patterns of autophagy–PANoptosis dysregulation presented in the subsequent sections.

### 3.4. Controversies and Unresolved Debates in Autophagy–PANoptosis Crosstalk

Despite significant progress in defining the molecular connections between autophagy and PANoptosis, several key controversies remain unresolved. Addressing these is essential for establishing a coherent framework for how the two pathways interact across tissues and disease contexts.

First, the directionality of autophagy’s influence on PANoptosis is still a matter of intense debate. Multiple studies within innate immune cells demonstrate that autophagy suppresses PANoptosis by degrading inflammasome components such as AIM2 and NLRP3, as well as key PANoptotic effectors including RIPK1, RIPK3, MLKL, and caspase-8 [10,16,51,52,53,54]. Autophagy additionally limits mitochondrial ROS and DAMP release through mitophagy, thereby reducing NLRP3 activation [16,58,59,60]. However, other evidence suggests that under certain inflammatory or infectious conditions, autophagy may facilitate PANoptosis. For instance, autophagy has been shown to promote ZBP1-dependent PANoptosis during viral infection [57], and autophagy-mediated degradation of c-FLIP or other anti-apoptotic factors can enhance caspase-8 activation, lowering the threshold for PANoptosis initiation. These opposing findings likely reflect differences in basal autophagy capacity, cytokine milieu, and metabolic status (e.g., mTOR–AMPK) across cell types [8,10,16,51].

Second, the bidirectional crosstalk between caspase-8 and autophagy remains incompletely understood. Caspase-8 can cleave essential autophagy proteins such as ATG3 and ATG5, thereby limiting autophagic flux [70,71,72,73,74,91]. At the same time, autophagy targets Caspase-8 itself, as well as upstream PANoptotic regulators including RIPK3 and ZBP1, for degradation [54,86]. Which of these interactions dominates appears to be highly context-dependent, varying substantially between macrophages, dendritic cells, neutrophils, and epithelial cells. This reciprocal regulation complicates attempts to define Caspase-8 as strictly pro- or anti-autophagic, or autophagy as strictly inhibitory or permissive for PANoptosis.

Third, mitophagy-dependent regulation of PANoptosis shows conflicting results across tissues. In macrophages and epithelial tissues, defective mitophagy consistently increases mtROS and mtDNA burden, promoting NLRP3 activation and PANoptotic signaling [16,64,88,89,90]. Yet, in certain macrophage models, mitophagy deficiency can have divergent effects depending on the infectious or cytokine environment. These discrepancies point to the need for standardized models capable of disentangling tissue-specific metabolic and immunological determinants of mitophagy–PANoptosis crosstalk.

Lastly, the function of p62/SQSTM1 is still up for debate. Although p62 can facilitate the clearance of ubiquitinated inflammasome components and thereby restrain PANoptotic signaling, p62 accumulation under impaired autophagy may act as a scaffold that enhances inflammasome activation. Moreover, as an adaptor for LC3, p62 links selective autophagy to inflammatory signaling pathways regulated by NF-κB and type I interferons [42,143], creating additional complexity in defining its net role across different disease states.

These unresolved debates call attention to the need for unified experimental frameworks and for interpreting findings within the specific metabolic, infectious, and immunological context of each model. The unified mechanistic model proposed in Section 3.3. provides a conceptual framework for reconciling these contradictions, but substantial gaps remain—particularly concerning the conditional switches that determine when autophagy restrains versus promotes PANoptosis.

## 4. Unraveling the Link Between Autophagy and PANoptosis in Immune-Mediated Inflammatory Diseases

Immune-mediated inflammatory diseases (IMIDs) are a category of chronic conditions marked by immune system dysregulation, resulting in ongoing inflammation that affects several tissues and organs [144]. Despite differing underlying origins, IMIDs have shared pathogenic pathways that involve innate and/or adaptive immunological responses, cytokine imbalances, and the breakdown of immune tolerance. This diverse cohort encompasses traditional autoimmune diseases (e.g., systemic lupus erythematosus, rheumatoid arthritis), autoinflammatory disorders (e.g., familial Mediterranean fever), and various inflammatory conditions characterized by mixed or ambiguous immunopathology (e.g., inflammatory bowel disease, psoriasis, multiple sclerosis). IMIDs are typically systemic, complex, and characterized by overlapping clinical and immunological characteristics [144].

**Figure 2:**
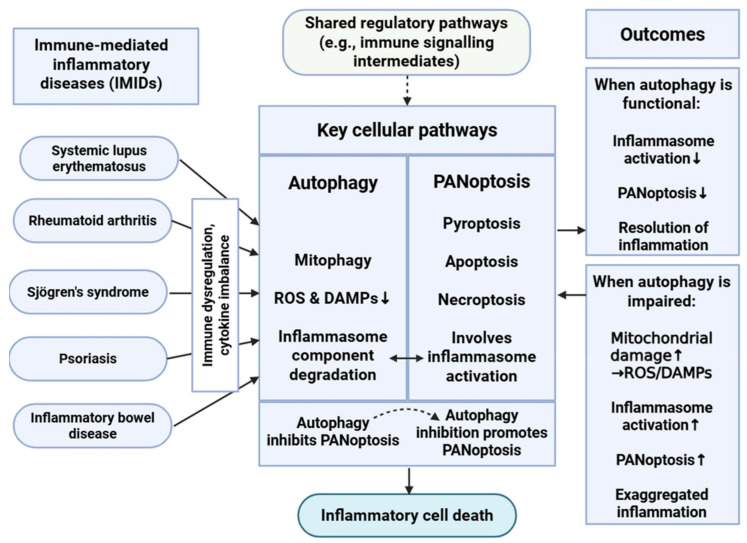
Interplay Between Autophagy and PANoptosis in Immune-Mediated Inflammatory Diseases. This schematic illustrates the complicated relationship between autophagy and PANoptosis in immune-mediated inflammatory diseases (IMIDs). Autophagy, particularly mitophagy, mitigates cellular stress by reducing reactive oxygen species (ROS) and danger-associated molecular patterns (DAMPs) and degrading inflammasome components, thereby inhibiting PANoptosis. Conversely, impaired autophagy leads to increased mitochondrial damage and inflammasome activation, promoting PANoptotic cell death and exacerbating inflammation. This dynamic balance plays a critical role in regulating immune responses and maintaining immune homeostasis in IMIDs. ↑: represents intensification or overactivation; ↓: represents decrease or decrease in function; dashed arrow represents indirect effect. Figure was partly created with https://biorender.com/ (accessed on 2 November 2025).

The interplay between PANoptosis and autophagy in IMIDs is a burgeoning field of study (Figure 2) [10,16]. Both are essential cellular mechanisms that significantly influence inflammation, apoptosis, and immunological responses—fundamental aspects of disease etiology.

The interplay between PANoptosis and autophagy represents a complex regulatory network with major consequences regarding immune homeostasis and inflammation [8,10,16,51,64]. Mitophagy limits NLRP3 activation by clearing damaged mitochondria, macroautophagy mediates the degradation of NLRP3 inflammasomes, and autophagy reduces the levels of ROS and DAMPs, both of which are potent activators of PANoptotic cell death pathways. In this context, autophagy acts as a negative regulator of PANoptosis, contributing to the resolution of inflammation and the maintenance of cellular integrity.

Conversely, inhibition or dysfunction of autophagy can enhance PANoptosis, leading to exaggerated inflammatory responses [8,10,16,51]. The loss of autophagic control may result in the accumulation of cellular stress signals, thereby promoting the assembly and activation of the PANoptosome complex. This relationship demonstrates the value of autophagy in modulating the threshold and extent of inflammatory cell death.

Moreover, PANoptosis and autophagy share several upstream regulatory pathways that highlight the integrated nature of these processes within the broader immune signaling network [10,16,51]. Autophagy can influence immune responses not only by clearing intracellular pathogens but also by degrading intermediates of immune signaling cascades, thereby indirectly modulating PANoptosome assembly and activation.

Inflammasome regulation serves as another critical point of intersection between these pathways [8,51]. PANoptosis frequently involves activation of inflammasomes, which are sensitive to mitochondrial dysfunction and oxidative stress. Autophagy, particularly mitophagy, can attenuate inflammasome activation by removing damaged mitochondria and mitigating the release of proinflammatory mediators. This inhibitory effect further illustrates how autophagy functions as a gatekeeper against uncontrolled inflammatory cell death, reinforcing its essential role in maintaining immune equilibrium [8,10,16,51].

Mechanistic perspective on disease-specific differences

Although the diseases discussed in this section are often described primarily in terms of autophagy deficiency or inflammasome overactivation, such descriptions underrepresent the mechanistic complexity underlying autophagy–PANoptosis interactions. A key determinant is how disease-specific microenvironments alter the thresholds defined in our unified model: type I IFN priming, cytokine burden, metabolic dysfunction, and genetic variants (e.g., *ATG16L1*, *NOD2*) shape whether autophagy restrains or amplifies PANoptosis. Therefore, each disease should be interpreted not only by its dominant inflammatory phenotype but also by how its molecular context shifts the balance between autophagic buffering and PANoptotic activation.

### 4.1. Systemic Lupus Erythematosus

Systemic lupus erythematosus (SLE) is a complex autoimmune disease marked by a loss of tolerance to self-antigens, leading to the production of autoantibodies. An overactive immune system leads to the accumulation of immunological complexes in tissues and organs, resulting in damage [145]. Recent transcriptomic analyses identified a PANoptosis-associated signature in SLE—ZBP1, MEFV, LCN2, IFI27, and HSP90AB1—across PBMCs [146,147,148,149,150,151]. Among immune cells, neutrophils and plasmacytoid dendritic cells (pDCs) provide the strongest experimental evidence for PANoptosis activation, with combined caspase-1, -8 and RIPK3 engagement [152,153,154,155,156,157]. T and B lymphocytes exhibit an upregulation of PANoptosis-related genes, although functional activation remains inferred [158,159,160,161,162,163].

The autophagy–PANoptosis interaction is particularly relevant in SLE because autophagy impairment amplifies the type I IFN response, increases mitochondrial ROS, and enhances ZBP1 expression—directly lowering the threshold for PANoptotic activation in neutrophils and pDCs [42,143,161,162,163].

Dysfunctional mitophagy leads to the persistence of oxidized mitochondrial DNA, which simultaneously serves as an autophagy-resistant DAMP and an upstream activator of inflammasomes, providing a disease-specific mechanistic bridge between defective autophagy and PANoptosis.

The strongest functional evidence for PANoptotic signaling in SLE derives from ex vivo human neutrophils and plasmacytoid dendritic cells, whereas some upstream mechanistic links have been modeled in murine lupus-prone strains.

**Figure 3:**
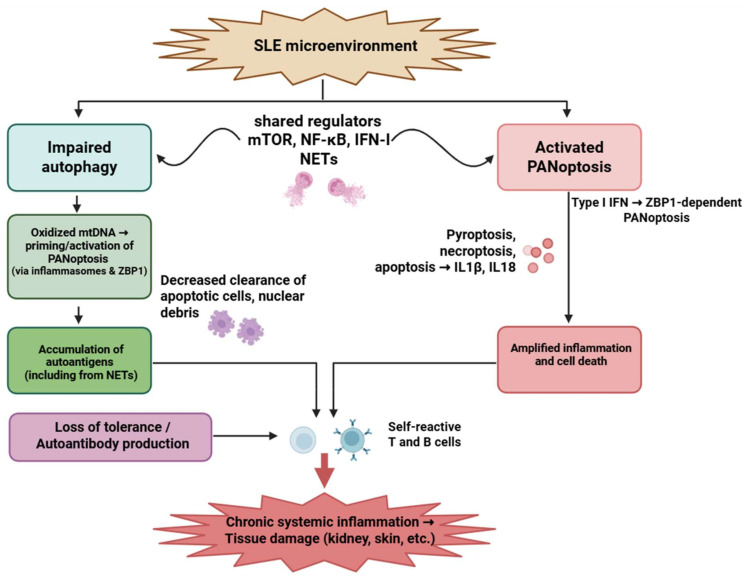
Interplay between impaired autophagy and PANoptosis in systemic lupus erythematosus. Autophagic dysfunction and aberrant PANoptotic activation led to the accumulation of autoantigens, exaggerated inflammatory responses, and tissue damage in SLE. Neutrophils, T cells, and B cells are critically involved, with overlapping signaling pathways such as mTOR, NF-κB, and type I interferons modulating both processes. Figure was partly created with https://biorender.com/ (accessed on 29 November 2025). mtDNA: mitochondrial deoxyribonucleic acid; ZBP1: Z-DNA-binding protein 1; NET: neutrophil extracellular trap; mTOR: Mechanistic target of rapamycin; NF-kB: nuclear factor-kB; IFN: interferon; IL: interleukin; SLE: systemic lupus erythematosus.

This IFN-high, mitochondrial-DNA–rich microenvironment is unique to SLE and is not observed in RA or psoriasis to the same extent, making ZBP1-dependent PANoptosis a particularly SLE-specific susceptibility factor. Targeting this interplay may offer new opportunities for therapeutic intervention aimed at restoring immune tolerance and limiting tissue damage (Figure 3).

### 4.2. Rheumatoid Arthritis

Rheumatoid arthritis (RA) is a chronic autoimmune disorder that can progressively impair tissues and organs beyond the joints, impacting the heart, liver, kidneys, and skin [164]. The relationship between PANoptosis and autophagy in RA is multifaceted and likely depends on the disease stage, inflammatory milieu, and specific immune cell populations involved.

In RA, PANoptosis is primarily observed in synovial macrophages and fibroblast-like synoviocytes (FLSs), where TNF and IL-1β exposure triggers caspase-8–RIPK3–NLRP3–dependent inflammatory cell death [165,166,167,168,169,170]. PANoptosis in lymphocytes remains inferred.

Autophagy in RA synovial cells normally restrains mitochondrial stress and inflammasome activation; however, chronic TNF-α exposure suppresses autophagic flux, promoting ROS accumulation and sensitizing macrophages and FLSs to PANoptosis [31,32,33,34,36,167,168,169,170].

Thus, while autophagy initially acts as a compensatory mechanism to limit inflammation, persistent cytokine stimulation drives a shift toward PANoptotic cell death—representing a disease-specific “autophagy exhaustion→PANoptosis amplification” pattern.

**Figure 4:**
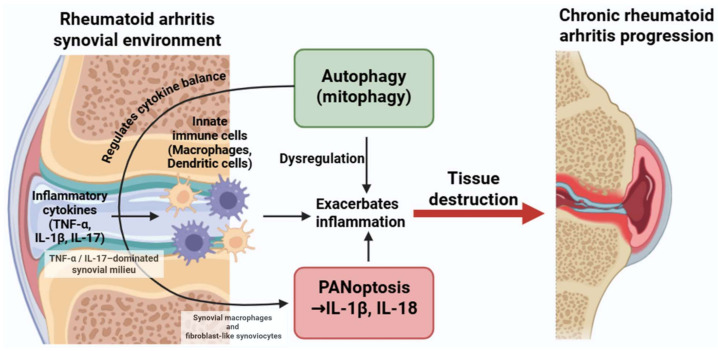
Dysregulated crosstalk between autophagy and PANoptosis in rheumatoid arthritis. Inflammatory cytokines such as TNF-α, IL-1β, and IL-17 activate both autophagic and PANoptotic pathways in macrophages and dendritic cells. While autophagy can have protective roles, its dysregulation may enhance PANoptosis and cytokine production, contributing to persistent inflammation and joint damage. Figure was partly created with https://biorender.com/ (accessed on 29 November 2025). TNF: tumor necrosis factor; IL: interleukin.

Unlike the IFN-dominated SLE environment, RA is characterized by a TNF-α/IL-17-driven metabolic and mitochondrial stress signature that uniquely modulates the autophagy–PANoptosis axis in synovial tissues. This establishes a pathological feedback loop wherein cytokine-driven autophagy and PANoptosis synergistically intensify inflammation and tissue destruction, contributing to disease progression (Figure 4). Most mechanistic insights into TNF- α or IL-1β–induced PANoptotic signaling in synovial cells have been obtained from human RA synovial tissue and cultured fibroblast-like synoviocytes, supplemented by murine inflammatory arthritis models.

### 4.3. Sjögren’s Syndrome

Sjögren’s syndrome (SS) is a systemic autoimmune and lymphoproliferative condition marked by unregulated lymphoplasmacytic infiltration in exocrine glands, including the salivary and lacrimal glands, leading to inflammation and tissue destruction. Excessively multiplying lymphocytes can potentially harm other organs, including the lungs, kidneys, and vascular walls [35].

PANoptosis in SS has been primarily inferred in salivary gland epithelial cells (SGECs), with activation of caspase-1, -8, and RIPK3 in response to ER stress and chronic inflammatory signaling [171,172,173,174,175,176,177]. Lymphocytic involvement remains hypothetical.

Autophagy in SGECs is markedly impaired, leading to ER stress accumulation, defective organelle clearance, and elevated release of DAMPs. These changes amplify inflammasome activation and promote a PANoptosis-permissive environment [173,174,175,176].

In contrast to other IMIDs, SGECs in SS demonstrate a unique convergence of impaired autophagy, ER stress, and lysosomal dysfunction, which together potentiate PANoptotic activation even in the absence of strong cytokine triggers [178,179].

**Figure 5:**
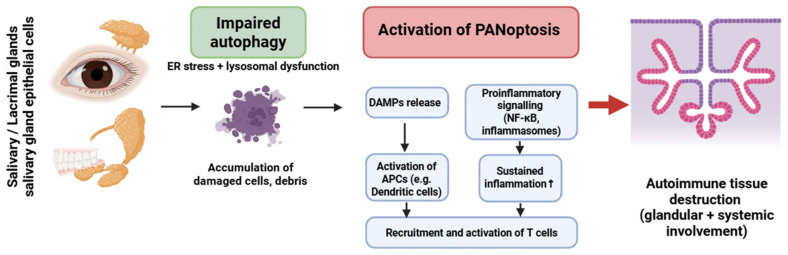
Crosstalk between impaired autophagy and PANoptosis in Sjögren’s syndrome. Dysfunctional autophagy in glandular epithelial cells promotes PANoptotic cell death, DAMP release, and inflammasome activation. This results in antigen-presenting cell activation and T-cell recruitment, leading to autoimmune inflammation and glandular tissue damage. The cycle is sustained by dysregulated NF-κB signaling and failure of tolerance mechanisms. Figure was partly created with https://biorender.com/ (accessed on 2 November 2025). ER: endoplasmic reticulum; DAMP: danger-associated molecular pattern; APC: antigen presenting cell; NF-kB: nuclear factor-kB.

This epithelial cell–centered autophagy defect distinguishes SS from RA (macrophage-dominant pathology) and SLE (IFN-driven neutrophil/pDC pathology) (Figure 5). Evidence for impaired autophagy and PANoptotic signaling in Sjögren’s syndrome primarily derives from human salivary gland epithelial cells, complemented by murine models of exocrine gland dysfunction.

### 4.4. Psoriasis

Psoriasis is an intricate, chronic, multifactorial inflammatory disorder characterized by the hyperproliferation of keratinocytes in the epidermis, resulting in an elevated epidermal cell turnover rate [180].

Psoriasis exhibits a unique PANoptotic pattern centered on keratinocytes and neutrophils. Lesional keratinocytes express a broad array of PANoptosis-related genes—*AIM2*, *CASP1*, *CASP4/5*, *GZMB*, *IL18*, *PYCARD*, *NOD2*, *STAT1*, and others—forming a molecular network distinct from other IMIDs [181,182,183,184,185,186,187,188,189,190,191,192]. Experimental models demonstrate AIM2-driven inflammasome activation and caspase-1/GSDMD-dependent cell death in keratinocytes, supporting partially demonstrated PANoptotic processes [180,181,182].

Neutrophils in psoriasis exhibit enhanced GSDMD-mediated inflammatory death, contributing to microabscess formation and fueling IL-17–driven inflammation—representing another experimentally confirmed PANoptosis-like pathway [39,115,116,117,118,119,120].

Autophagy defects in psoriasis differ from those in SLE or RA: keratinocytes exhibit reduced autophagic degradation of protein aggregates, altered lipid metabolism, and dysregulated mTOR activity, all of which sensitize them to PANoptotic signaling [180,193]. The dominant molecular signature is STAT1-IRF1–AIM2 axis activation, driven by IFN-γ and IL-17, which is not prominent in RA or SS.

Mechanistic data on AIM2-inflammasome activation and PANoptotic features in keratinocytes come largely from human psoriatic lesional skin, whereas neutrophil-associated GSDMD activation has been demonstrated in both human neutrophils and murine inflammatory models.

**Figure 6:**
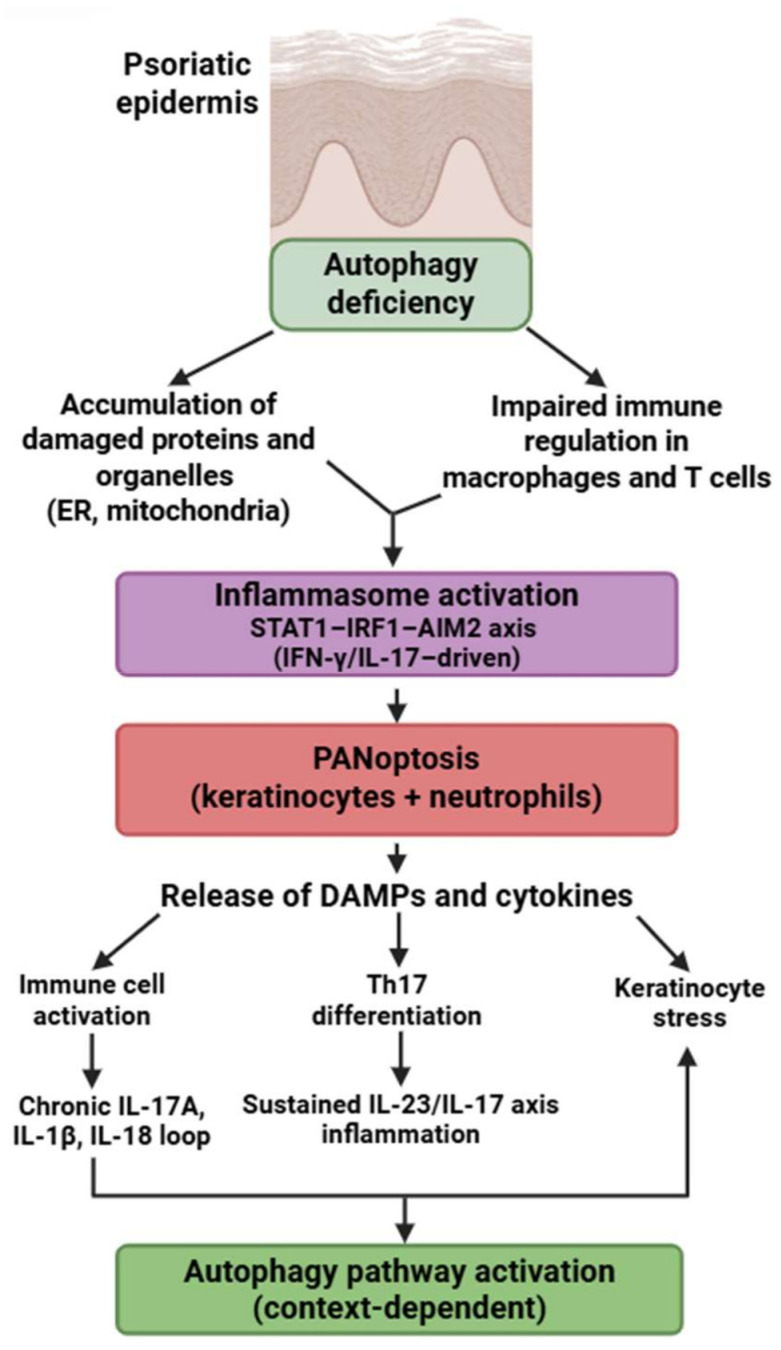
Autophagy-PANoptosis axis in psoriasis pathogenesis. Defective autophagy promotes organelle stress, inflammasome activation, and PANoptosis in keratinocytes and immune cells. PANoptotic cell death leads to the release of DAMPs and proinflammatory cytokines, enhancing Th17 differentiation and keratinocyte activation. This feed-forward loop sustains the chronic inflammatory environment characteristic of psoriasis. Figure was partly created with https://biorender.com/ (accessed on 2 November 2025). ER: endoplasmic reticulum; DAMP: danger-associated molecular pattern; IL: interleukin; STAT1: signal transducer and activator of transcription 1; IRF1: interferon regulatory factor 1; AIM2: absent in melanoma 2; IFN: interferon.

These mechanisms highlight that psoriasis is characterized not merely by hyperproliferative keratinocytes but by a distinctive, autophagy-impaired, AIM2-driven PANoptotic program. This process is further reinforced by robust PANoptosis in neutrophils and a STAT1/IRF1-high autoinflammatory signature (Figure 6).

### 4.5. Inflammatory Bowel Disease

Inflammatory bowel disease (IBD) is a group of chronic, relapsing inflammatory disorders of the gastrointestinal tract, primarily including Crohn’s disease and ulcerative colitis, caused by dysregulated immune responses to intestinal microbiota in genetically susceptible individuals [194]. In addition to gastrointestinal symptoms such as abdominal pain, diarrhea, and weight loss, IBD may present with extraintestinal manifestations affecting the skin, joints, eyes, hepatobiliary system, and other organs [195].

The strongest evidence for PANoptosis in IBD exists in intestinal epithelial cells (IECs), particularly in Crohn’s disease, where impaired function of ATG16L1, IRGM and NOD2 promotes NLRP3–caspase-1–caspase-8–RIPK3 activation [82,196,197]. Macrophage involvement is inferred.

Autophagy plays an essential protective role in IECs by preventing excessive inflammasome activation, clearing damaged mitochondria, and limiting DAMP release. Loss of ATG16L1-mediated autophagy directly facilitates PANoptosome assembly and sensitizes IECs to PANoptotic cell death [196].

Unlike SLE, where autophagy failure is IFN-driven, or RA, where cytokine overload suppresses autophagy, IBD is uniquely shaped by microbial PAMP–driven autophagy impairment, resulting in a distinctive microbiota-dependent PANoptotic phenotype.

**Figure 7:**
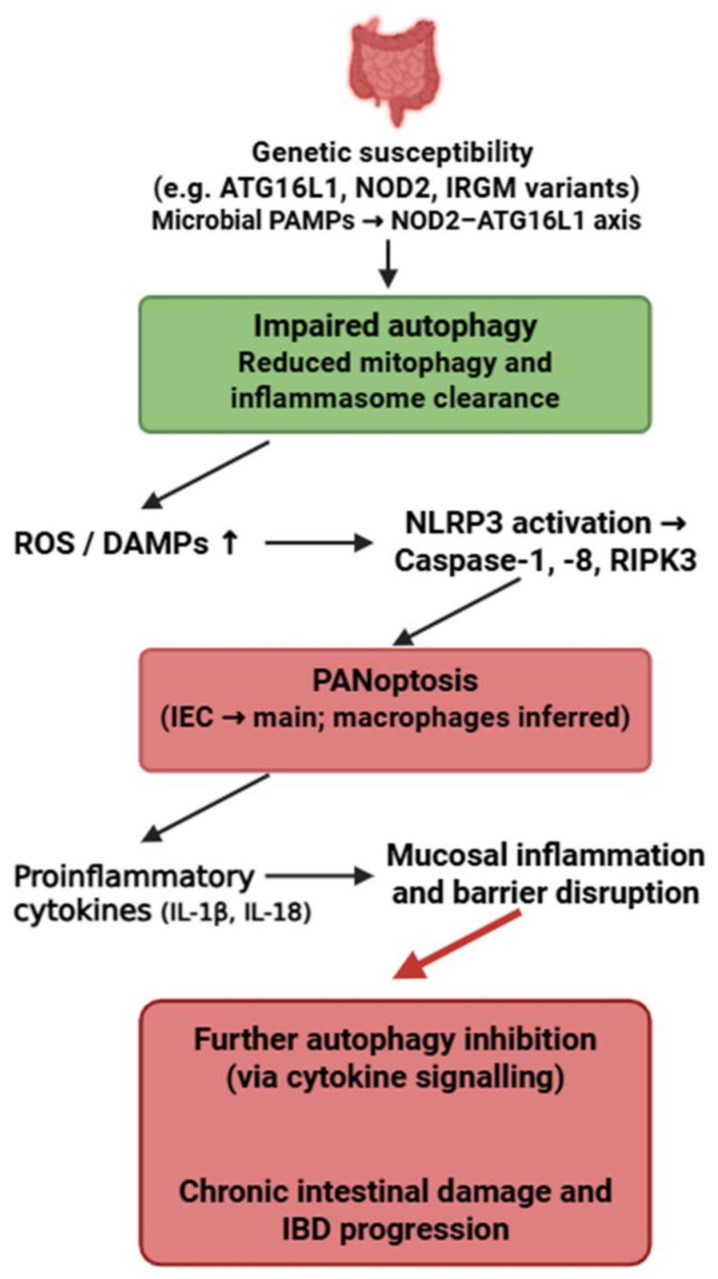
Bidirectional crosstalk between autophagy and PANoptosis in inflammatory bowel disease. Genetic variants affecting autophagy-related pathways impair the clearance of damaged mitochondria and inflammasome components, leading to increased ROS, DAMPs, and activation of PANoptosis. This inflammatory cell death further suppresses autophagy and exacerbates mucosal inflammation and intestinal barrier disruption, forming a pathological feedback loop driving IBD progression. Figure was partly created with https://biorender.com/ (accessed on 2 November 2025). PAMP: pathogen-associated molecular pattern; ATG16L1: Autophagy related 16 like 1; NOD2: Nucleotide Binding Oligomerization Domain Containing 2; IRGM: Immunity-Related GTPase M; ROS: reactive oxygen species; DAMP: danger-associated molecular pattern; NLRP3: NLR family pyrin domain containing 3; RIPK3: Receptor-interacting protein kinase 3; IEC: intestinal epithelial cell; IL: interleukin; IBD: inflammatory bowel disease.

Thus, the microbiota–NOD2–ATG16L1 axis represents a disease-specific upstream regulator of the autophagy–PANoptosis interaction in IBD. This bidirectional crosstalk highlights a pathogenic feedback loop between defective autophagy and PANoptosis that may play a pivotal role in the initiation and progression of IBD (Figure 7). While the genetic risk variants *ATG16L1-T300A, NOD2* and *IRGM* are established from human cohort studies, most functional evidence linking impaired autophagy to PANoptosis in intestinal epithelial cells arises from murine *Atg16l1-* or *Nod2*-deficient models.

#### Outstanding Challenges and Limits of Current Evidence

Across autoimmune, autoinflammatory, infectious, and malignant diseases, autophagy–PANoptosis interactions display both shared principles and striking divergences. However, most available data derive from models that differ in cell type, metabolic state, and cytokine exposure, limiting cross-study comparability. Many reported differences may reflect experimental context rather than true mechanistic contradictions. Future work must clarify tissue-specific thresholds, identify biomarkers that predict PANoptotic propensity, and standardize approaches to measuring autophagy flux in inflammatory settings. A central challenge remains determining whether PANoptosis-driving stimuli primarily overwhelm autophagy or whether dysregulated autophagy actively promotes PANoptosis in particular disease states. Addressing these gaps will be essential for translating the mechanistic insights synthesized in this review into therapeutic strategies.

## 5. Future Directions

Although considerable advances have been achieved in delineating PANoptosis, the relationship between autophagy impairment and PANoptotic cell death in IMIDs continues to be inadequately understood. Future investigations should focus on elucidating how particular autophagy pathways—specifically mitophagy, xenophagy, and ER-phagy—contribute to the modulation of susceptibility to PANoptosis among various immune and stromal cell types. Current evidence suggests that impaired autophagy enhances mitochondrial ROS production, DAMP release, and inflammasome activation, thereby reducing the threshold for PANoptosis [31,32,33,34,35,36]; however, the exact sequence and hierarchy of these events in vivo remain to be elucidated.

Another significant avenue involves the development of cell-type-specific models that facilitate the conditional regulation of autophagy-related genes (e.g., *Atg5*, *Atg7*, *Atg16l1*) in conjunction with PANoptosis mediators (*Zbp1*, *Ripk3*, *Casp1/8*). Such models will aid in elucidating whether autophagy functions predominantly as an upstream suppressor; a parallel regulator; or, in certain contexts, a facilitator of PANoptotic responses—an issue that remains unresolved in human IMIDs.

Furthermore, the identification of multi-omic biomarkers capable of differentiating autophagy-dependent from autophagy-independent PANoptotic states remains a significant unmet need. Integrating transcriptomic data with proteomic analysis and gasdermin cleavage profiling may enhance disease stratification and facilitate the identification of patients in whom the autophagy–PANoptosis axis is the primary driver of pathology [31,32,33,34,36]. Finally, future translational research should focus on elucidating the timing and mechanisms by which modulation of autophagy influences PANoptotic susceptibility in a therapeutically relevant manner. Because both the induction of autophagy and the inhibition of PANoptosis may compromise host defense mechanisms or redirect cells toward alternative programmed cell death pathways, the safety and context-dependent effects of targeting this axis must be thoroughly assessed [32,33,34,35,36].

Natural compounds such as baicalin have been shown to modulate autophagy (via PI3K/Akt/mTOR) and inhibit PANoptotic signaling (including ZBP1–RIPK3–NLRP3 pathways), highlighting their potential as context-dependent therapeutic modulators of inflammatory cell death [96,97].

Together, these forthcoming research priorities will be crucial for elucidating the mechanistic hierarchy connecting autophagy dysregulation to PANoptosis and for translating these insights into targeted, disease-specific therapeutic strategies.

## 6. Therapeutic Targeting of the Autophagy–PANoptosis Axis in Immune-Mediated Inflammatory Diseases

Therapeutic modulation of the autophagy-PANoptosis axis presents a promising emerging approach in the treatment of IMIDs, owing to the pivotal role of autophagy in mitigating mitochondrial stress, preventing DAMP release, and inhibiting inflammasome activation, all of which contribute to the induction of PANoptotic cell death. Numerous critical molecular nodes within this axis are already amenable to targeting through existing or investigational agents. Inhibition of the RIPK1–RIPK3 signaling complex, a central element of the PANoptosome, has demonstrated the ability to suppress necroinflammatory signaling and diminish downstream inflammatory cell death in preclinical models, underscoring its therapeutic potential, especially in environments rich in TNF and IL-17, such as RA and psoriasis [31,32]. Similarly, pharmacological modulation of caspase-1 and caspase-8 can diminish inflammasome-mediated cytokine secretion and pyroptosis, thereby indirectly inhibiting PANoptosis. Caspase-1 inhibitors, such as VX-765, have shown effectiveness in reducing IL-1β-mediated inflammation, indicating potential significance for conditions like SLE and IBD where inflammasome activation plays a key pathogenic role [31,33].

An additional promising approach entails augmenting autophagic flux to reestablish mitochondrial homeostasis and diminish vulnerability to PANoptosis. Compounds including rapamycin, metformin, spermidine, and AMPK activators have demonstrated the ability to enhance mitophagy and suppress inflammatory signaling, consequently reducing PANoptotic activation in experimental models [35]. This approach may be especially advantageous in conditions marked by significant autophagy impairment, such as SLE—where defective mitophagy facilitates mtDNA-induced PANoptosis—and SS or IBD, where epithelial autophagy deficiencies enhance inflammasome activation and tissue damage.

Therapies targeting inflammasomes also intersect with the PANoptosis pathway. Inhibitors of NLRP3, such as MCC950-like compounds, have been demonstrated to inhibit caspase-1 activation and gasdermin-mediated cell death, consequently attenuating inflammatory tissue injury across various experimental models. These agents may provide therapeutic advantages for conditions such as RA, psoriasis, and IBD, wherein increased inflammasome activation intensifies local inflammatory responses [31,34]. Furthermore, modulation of the ZBP1–type I interferon axis provides a targeted therapeutic strategy, especially in the treatment of SLE. ZBP1 functions as an upstream nucleic acid sensor that orchestrates the activation of RIPK3, caspase-8, and the inflammasome; consequently, therapeutic strategies that diminish type I IFN signaling may indirectly reduce ZBP1-dependent PANoptotic priming [31,36].

Although these strategies hold significant potential, it is essential to recognize important safety considerations. Because PANoptosis encompasses pathways vital for antiviral defense, immunosurveillance, and coordinated inflammatory responses, extensive inhibition may elevate vulnerability to infections or redirect cell death toward alternative, potentially deleterious pathways such as necroptosis or pyroptosis [32,33,34,36]. Similarly, systemic augmentation of autophagy may influence metabolic regulation or tissue regeneration in unforeseen manners, emphasizing the importance of meticulously calibrated, context-specific strategies. Overall, although therapeutic modulation of autophagy and PANoptosis is scientifically persuasive, its effective clinical implementation will necessitate precise, disease-specific strategies, biomarker-driven patient selection, and comprehensive assessment of long-term safety.

## 7. Conclusions

The interplay between autophagy and PANoptosis has emerged as a central regulator of immune cell fate and inflammatory responses. Rather than functioning as isolated pathways, autophagy and the PANoptosome form an interconnected axis in which autophagy constrains inflammatory cell death but can also, in specific contexts, promote PANoptotic signaling. This balance is very dependent on the type of cell and the type of stress, and it is controlled by common regulators like ZBP1, RIPK3, caspase-8, and ATG proteins.

Across immune-mediated inflammatory diseases—including systemic lupus erythematosus, rheumatoid arthritis, Sjögren’s syndrome, psoriasis, and inflammatory bowel disease—disrupted autophagy is consistently associated with heightened PANoptotic activity. This convergence drives chronic inflammation, loss of immune tolerance, and tissue injury, underscoring the autophagy–PANoptosis axis as a unifying mechanism in IMID pathogenesis.

These insights also highlight new therapeutic opportunities. Interventions that restore autophagic flux or modulate PANoptosome activation may allow more precise control of inflammatory responses. However, because the interaction between autophagy and PANoptosis is highly context-dependent, cell-type-specific and disease-specific understanding will be essential before such strategies can be safely translated.

Ultimately, viewing the autophagy–PANoptosis interaction as a core immune regulator offers a framework to inspire future research and support the creation of targeted treatments for chronic autoimmune and autoinflammatory conditions.

## Figures and Tables

**Table 1:** The context-dependent outcomes of autophagy and PANoptosis cooperation. PAMP: pathogen-associated molecular patterns; DAMP: danger-associated molecular patterns; ATG: autophagy-related; ZBP1: Z-DNA-binding protein 1; ROS: reactive oxygen species; NLRP3: NOD-, LRR- and pyrin domain-containing protein 3.

Condition	The Effect of Autophagy	The Outcome of PANoptosis
Normal conditions	Autophagy clears inflammatory signals [10,37,63]	PANoptosis is inhibited [16]
Infection, PAMP/DAMP	Autophagy regulates the strength of the response [10,16,63,65]	PANoptosis is triggered when there is too much stimulus [66,67,68,69]
Genetic defect (ATG mutation)	Autophagy is dysfunctional [77,78,79,80,81,82,83,84,85]	Inflammasome/PANoptosome activation [3,79,80,81,82,83,84,85]
Type I interferon environment	Autophagy deficiency amplifies ZBP1-dependent PANoptosis [66,67,68,69]	Prone to PANoptosis [16,66,67,68,69]
Mitochondrial stress	Mitophagy is overloaded or insufficient [16,64]	ROS→NLRP3 activation→PANoptosis [16,64]

**Table 2:** The functions of autophagy and PANoptosis in innate immune cells. *: Mixed inflammatory cell death suggestive of PANoptosis, but not yet mechanistically proven. TLR: Toll-like-receptor; ROS: reactive oxygen species; IL: interleukin; LAP: LC3-associated phagocytosis; NK: natural killer; FoxO1: Forkhead box protein O1; LPS: lipopolysaccharide; IFN: interferon; ZBP1: Z-DNA-binding protein 1.

Cell Types	Functions of Autophagy	PANoptosis Activation
Macrophage	Mitophagy, pathogen elimination [80,86,87,88,97]	LPS + IFNγ: ZBP1-dependent PANoptosis [22,41,64,86,88,90]
Dendritic cell	Antigen presentation, TLR-regulation [98,99]	TLR-activation can lead to PANoptosome formation [100,101,102]
Monocyte	ROS, IL-1β regulation [13,62,103,104,105,106,107]	Increased inflammasome/PANoptosis in inflammation [108,109]
Neutrophil	Lipophagy, xenophagy, and LAP connected to NETosis [110,111,112,113,114]	Gasdermin D and caspase-8 can lead to PANoptotic-like* death [39,115,118,119,120]
NK cell	FoxO1-mediated autophagy during NK cell formation and function [121]	Limited evidence; mechanistic PANoptosis links remain speculative

**Table 3:** Autophagy—PANoptosis interaction in adaptive immune cells. IFN: interferon; SLE: systemic lupus erythematosus; RA: rheumatoid arthritis; ZBP1: Z-DNA-binding protein 1; RIPK3: Receptor-interacting protein kinase 3; NLRP3: NOD-, LRR- and pyrin domain-containing protein 3.

Context	Role of Autophagy	PANoptosis Potential
Naïve T cells survival	Maintains metabolic stability and viability [122,124]	Low risk
Activated T cells (e.g., IFN-I, virus)	Regulates oxidative stress and survival [125,126,132]	ZBP1, Caspase-8, RIPK3 activation possible under inflammatory stress [30,135,136,137,138]
Memory T cells	Ensures mitochondrial quality control (mitophagy) [133,134]	Vulnerable under chronic stress or impaired autophagy [132,133,134,135]
B cell activation	Supports antigen presentation and survival [139]	Inflammasome and death complex components may be activated [141,142]
Autoimmune settings (e.g., SLE, RA)	Autophagy is overwhelmed or dysfunctional [16]	PANoptosis may be triggered via ZBP1, NLRP3, Caspase-8 pathways [16,135,136]
Tumor-infiltrating lymphocytes	Autophagy exhausted in chronic inflammation [16,129]	High expression of PANoptosome components possible [129,130,131]

## Data Availability

No new data were created.

## Abbreviations

The following abbreviations are used in this manuscript:

AIM2	Absent in Melanoma 2
AMP-AMPK	Adenosine Monophosphate-activated protein kinase
ASC	apoptosis-associated speck-like protein containing a CARD
ATGs	autophagy-related genes
BAK1	BRI1-associated receptor kinase 1
CASP	caspase
CYCS	Cytochrome c
DAMPs	damage-associated molecular patterns
DCs	dendritic cells
ER	endoplasmic reticulum
FoxO1	Forkhead box protein O1
GSDM	gasdermin
GZMA/B	Granzyme A/B
HSP90AB1	Heat Shock Protein 90 Alpha Family Class B Member 1
HSPA4	Heat Shock Protein Family A (Hsp70) Member 4
IBD	Inflammatory bowel disease
IEC	intestinal epithelial cell
IFI27	Interferon Alpha Inducible Protein 27
IFN	interferons
IL	interleukin
IMIDs	Immune-mediated inflammatory diseases
iNKs	immature NK cells
IRF1	Interferon Regulatory Factor 1
IRGM	immunity-related GTPase M
LC3	microtubule-associated protein 1 light chain 3
LCN2	Lipocalin-2
LPS	lipopolysaccharide
MAPK	Mitogen-Activated Protein Kinase
MEFV	Mediterranean Fever
MHC	major histocompatibility complex
MLKL	mixed lineage kinase domain-like pseudokinase
mTOR	mechanistic target of rapamycin
NET	neutrophil extracellular trap
NF-κB	Nuclear Factor kappa B
NK	natural killer
NLRP3	NLR family pyrin domain-containing protein 3
NOD2	Nucleotide-binding oligomerization domain-containing protein 2
p62/SQSTM1	protein sequestosome 1
PAMPs	pathogen-associated molecular patterns
PARP	Poly (ADP-ribose) polymerase
PBMCs	Peripheral Blood Mononuclear Cells
PI3K/Akt	Phosphoinositide 3-kinase / Protein kinase B
pMLKL	phosphorylated mixed lineage kinase domain-like pseudokinase
PYCARD	PYD And CARD Domain Containing
RA	Rheumatoid Arthritis
RIPK3	receptor-interacting serine/threonine-protein kinase 3
ROS	reactive oxygen species
S100A12	S100 Calcium Binding Protein A12
SLE	Systemic Lupus Erythematosus
STAT1	Signal Transducer and Activator of Transcription 1
TLR	Toll-like receptor
TNF	tumor necrosis factor
ULK1	Unc-51 like autophagy activating kinase 1
Vps34	vacuolar protein sorting 34
ZBP1	Z-DNA binding protein 1
